# Fluctuations at a Low Mean Temperature Accelerate Dengue Virus Transmission by *Aedes aegypti*

**DOI:** 10.1371/journal.pntd.0002190

**Published:** 2013-04-25

**Authors:** Lauren B. Carrington, M. Veronica Armijos, Louis Lambrechts, Thomas W. Scott

**Affiliations:** 1 Department of Entomology, University of California Davis, Davis, California, United States of America; 2 Insects and Infectious Diseases, CNRS URA 3012, Institut Pasteur, Paris, France; 3 Fogarty International Center, National Institutes of Health, Bethesda, Maryland, United States of America; United States Army Medical Research Institute of Infectious Diseases, United States of America

## Abstract

**Background:**

Environmental factors such as temperature can alter mosquito vector competence for arboviruses. Results from recent studies indicate that daily fluctuations around an intermediate mean temperature (26°C) reduce vector competence of *Aedes aeygpti* for dengue viruses (DENV). Theoretical predictions suggest that the mean temperature in combination with the magnitude of the diurnal temperature range (DTR) mediate the direction of these effects.

**Methodology/Principal Findings:**

We tested the effect of temperature fluctuations on *Ae. aegypti* vector competence for DENV serotype-1 at high and low mean temperatures, and confirmed this theoretical prediction. A small DTR had no effect on vector competence around a high (30°C) mean, but a large DTR at low temperature (20°C) increased the proportion of infected mosquitoes with a disseminated infection by 60% at 21 and 28 days post-exposure compared to a constant 20°C. This effect resulted from a marked shortening of DENV extrinsic incubation period (EIP) in its mosquito vector; i.e., a decrease from 29.6 to 18.9 days under the fluctuating *vs.* constant temperature treatment.

**Conclusions:**

Our results indicate that *Ae. aegypti* exposed to large fluctuations at low temperatures have a significantly shorter virus EIP than under constant temperature conditions at the same mean, leading to a considerably greater potential for DENV transmission. These results emphasize the value of accounting for daily temperature variation in an effort to more accurately understand and predict the risk of mosquito-borne pathogen transmission, provide a mechanism for sustained DENV transmission in endemic areas during cooler times of the year, and indicate that DENV transmission could be more efficient in temperate regions than previously anticipated.

## Author Summary

Mosquitoes in the wild are exposed to daily fluctuations in temperature, but in the laboratory, the effect of temperature on vector competence is generally assessed using constant temperatures. Recent studies demonstrate that realistic fluctuations in temperature around an intermediate mean (26°C) can alter life-history traits, population dynamics, and the ability of a mosquito to become infected with and transmit dengue virus (DENV). Here we tested how fluctuations around high and low mean temperatures influence vector competence and the extrinsic incubation period. Small fluctuations around a high mean temperature (∼8°C swings around 30°C) had no detectable effect on vector competence. Large fluctuations around a low mean (∼18°C swings around 20°C) demonstrate that only 18.9 days were required for 50% of DENV-exposed mosquitoes to develop a disseminated infection, compared to 29.6 days at constant 20°C. Twenty-eight days post-exposure to the infectious blood meal, 100% of mosquitoes tested had a disseminated infection under fluctuating temperatures, but under a constant temperature this proportion was only 42%. Reduced duration of extrinsic incubation increases the potential for pathogen transmission. Results indicate that the rate of dengue transmission by mosquitoes in temperate regions with natural fluctuations may be underestimated by experiments conducted under constant temperatures.

## Introduction

The ability of *Aedes aegypti* to transmit viruses, in particular dengue viruses (DENV), has long been known to be influenced by temperature [1]–[6]. It is generally assumed that higher mean temperatures facilitate DENV transmission due to faster virus propagation and dissemination within the vector. Vector competence, the probability of a mosquito becoming infected and subsequently transmitting virus after ingestion of an infectious blood meal [7], is generally positively associated with temperature, whereas the duration of the virus extrinsic incubation period (EIP) associates negatively with temperature [6].

The norms of reaction (i.e., phenotypic variation across environmental variation) of vector competence and EIP have been well documented for a large range of temperatures for *Ae. aegypti*. At high temperatures (26°C and above), DENV dissemination and transmission can be observed in one week or less [5], [6], [8], [9]. Lower temperatures generally extend the duration of EIP [5], [6], [8]; at 21°C and below, the EIP for DENV can be in the order of several weeks [3], [4]. Despite these prolonged incubation periods, DENV-infected *Ae. aegypti* are capable of transmitting virus under laboratory conditions after incubation at temperatures as low as 13°C [4], and can become infective after incubation under temperature as low as 10°C [8]. Evidence to support an upper thermal threshold for DENV transmission is more limited. There is a well-established link between temperature and many of the life-history traits of *Ae. aegypti*, with a (population dependent) thermal optimum for development, reproduction and survival [10]. Beyond this, subsequent increases in temperature become detrimental for the mosquito; i.e., immature development rate slows as mortality increases, adult reproductive function is impaired in the high 30°'s, and adult survival declines as temperature continues to rise [11], [12]. *Ae. aegypti* vector competence for DENV has been detected up to a maximum of 35°C [6], but at temperatures in excess of this, accurately measuring vector competence indices before the mosquito dies is difficult.

What is much less well-documented is the influence of fluctuations in daily temperature on the norm of reaction of vector competence and EIP. Indeed, environmental temperature under natural conditions does not remain constant, but oscillates between a minimum at night and a maximum during daytime. Results from studies using realistic fluctuating temperature profiles support the notion that fluctuating temperatures may alter estimates of both life history traits and vector competence of mosquitoes [9], [12]–[16], with the magnitude of the diurnal temperature range (DTR) associated with the degree of response observed. Vector competence of *Ae. aegypti* for DENV examined under fluctuating temperatures, indicated that a large DTR of ∼20°C around an intermediate mean of 26°C (i.e., ∼16°C to 36°C; temperatures representative of conditions mosquitoes in central west Thailand would be exposed to in the low DENV transmission season) reduced the proportion of *Ae. aegypti* females with a midgut infection and reduced female survival. At a mean of 26°C, EIP did not vary if temperature fluctuations were symmetric whereas EIP tended to last longer under more natural asymmetric fluctuations [9], [13].

While the effects of realistic temperature fluctuations on *Ae. aegypti* vector competence and EIP for DENV at an intermediate mean temperature (26°C) have recently been described [9], [13], [14], the impact of fluctuations at the upper and lower thermal limits are unknown. Short periods of the day spent at extreme temperatures may affect key steps of the mosquito infection process. Evidence suggests that DENV transmission may be more limited by lower daily temperatures [17], as opposed to average daily temperatures.

In this study we investigated whether fluctuations at high and low mean temperatures alter adult survival, vector competence and/or EIP of *Ae. aegypti* for DENV serotype-1 (DENV-1), compared to constant temperatures. We then explored how this might affect the geographical range of DENV in light of our understanding of the thermal limits of DENV transmission. Based on theoretical predictions [9], we expect that large fluctuations at low temperatures will enhance transmission (increase infection/dissemination probability and reduce the EIP of the virus) because of time spent under warmer (more optimal) conditions, whereas fluctuations at high temperatures will have a negative effect because of time spent at elevated temperatures detrimental to the vector and/or the virus. To test this hypothesis, we exposed mosquitoes to high and low temperatures with and without fluctuations across two experiments, and assayed mosquitoes for virus infection.

## Methods

### Experimental design

We determined the effect of constant and fluctuating temperature regimes at both high and low mean temperatures, on the survival and vector competence of *Ae. aegypti* for DENV-1. Over the course of two experiments we tested seven temperature regimes. At the low temperatures, we exposed mosquitoes to three constant temperatures (16°C, 20°C and 26°C) and one fluctuating temperature regime (a DTR of 18.6°C around a mean of 20°C). The minimum programmed temperature for the fluctuations was 11.7°C and the maximum was 30.3°C. Given the low temperatures in this experiment and the associated uncertainty of whether we would identify any infection, we included the 26°C treatment as a control temperature, knowing we could detect DENV infected females at this temperature. At the upper end of the temperature scale, we tested two constant temperature regimes (30°C and 35°C), and one cyclic temperature regime with a DTR of 7.6°C. Temperatures fluctuated between 27.1°C and 34.7°C, around a mean of 30°C. We included the 35°C constant temperature treatment to ensure that the peak temperature was not a limiting factor of infection potential.

The magnitude and asymmetrical shape of the temperature profiles were based on temperature recordings from Central West Thailand where DENV is endemic [14]. Fluctuating temperature regimes followed a sinusoidal progression during the day, and a negative exponential decrease at night, with minimum and maximum temperatures reached at 06:00 and 14:00 respectively. A 12∶12 hr light∶dark cycle was used, with the light schedule changing at 08:00 and 20:00. Experimental mosquitoes were housed in KBF115 incubators (Binder, Tuttlingen, Germany) that maintained climatic conditions. HOBO data loggers (Onset, Cape Cod, MA) recorded temperatures on an hourly basis in the two incubators with fluctuations. Actual air temperatures within the incubators followed the programmed temperature profile closely. There was an average of <0.3°C difference between the daily programmed temperature and the actual air temperature inside the incubators across treatments. Relative humidity was maintained between 70% and 80% across all treatments, and was also recorded by data loggers.

### Mosquitoes

*Ae. aegypti* used in our experiments were collected from Kamphaeng Phet Province, Thailand as pupae during January 2011 and sent to UC Davis as F_1_ eggs. After eggs were received, they were hatched and reared at a low density (1.3 larvae/10 mL) in 24 cm×29 cm×5 cm containers with 1.5 L of deionized water. Colony maintenance was conducted under standard insectary conditions (constant 28°C±2°C, 70–80% RH) and a 12∶12 hr light∶dark cycle, with >500 females per generation. Larvae were fed a 1∶1 mix of bovine liver powder and puppy chow, with 0.1 g per 200 larvae each day for the first four days, 0.2 g on the fifth day, 0.3 g on the sixth, and then 0.2 g on the remaining two days, at which time most larvae had pupated. Generation F_4_ mosquitoes used in experiments.

### Experimental infections

When females were 4–5 days old, access to sucrose was removed for 24–36 hr, after which time females were fed defibrinated sheep blood (QuadFive, Ryegate, MT), mixed with DENV-1 freshly grown in cell culture prior to mosquito exposure, using an artificial feeding system. Virus supernatant was harvested after scraping and then separating all cells by centrifugation. Mosquitoes were fed through a desalted porcine intestinal membrane stretched over the bottom of a warm water-filled jar to maintain a temperature of 37°C. The viral isolate used, SV2951 obtained from Ratchaburi, Thailand, had been passaged at 28°C seven times in *Ae. albopictus* C6/36 cells prior to use in this study. While this is potentially sufficient time for adaptation to cell culture temperatures, we do not consider it likely that this would influence our results as 28°C is not deemed as a stressful temperature for DENV. Confluent cultures of C6/36 cells grown in 25-cm^2^ flasks were inoculated at a virus multiplicity of infection of 0.01 and left to grow for 10 days at 28°C in 5% CO_2_. The infectious blood meal consisted of 50% defibrinated sheep blood (Quadfive, MT), 45% viral supernatant harvested at Day 10, and 2.5% sucrose solution (diluted 1∶4 in water) and 2.5% adenosine triphosphate disodium salt (Sigma-Aldrich, MO) at a final concentration of 5×10^−3^ M.

We prepared one blood meal for each experiment. The blood meal for the low temperature experiment was calculated to contain 5.86×10^5^ focus forming units (FFU)/ml of DENV-1. The calculated titer for the high temperature experiment was 7.89×10^5^ FFU/ml. Mosquitoes in both experiments were limited to 35 min feeding, to minimize the effect of virus degradation in the infectious blood meal. Mosquitoes were allowed 2–3 hr to begin digestion after the blood meal. We subsequently sedated them using CO_2_ and retained only fully engorged females to set up experimental groups. For the low temperature experiment, forty-four replicate 1-pint paper cartons (Science Supplies WLE, NJ) with mesh tops, each containing 20 engorged females were set up. Twelve cartons were placed into each of the experimental temperature regimes, and eight cartons into the control 26°C incubator. For the high temperature experiment, we tested 28 replicate cartons each containing 16 females. Nine cartons were placed into the constant temperature incubators, and 10 into the 30°C plus fluctuation incubator.

### Vector competence

We assessed vector competence at 7, 14, 21 and 28 days post exposure (DPE) to the infectious DENV-1 blood meal (i.e., days of EIP) for mosquitoes in the low temperature experiment. At each time point, we sampled three replicate cartons of mosquitoes from each experimental temperature, and two from the control 26°C treatment. At the high temperatures, mosquitoes were sampled at 3, 6 and 9 DPE. Three cartons were randomly removed from each incubator at each time point. The additional carton in the 30°C fluctuation treatment was also tested at 9 DPE. Because the course of DENV infection in the mosquito is faster at higher temperatures than at lower ones [4], [6], we sampled mosquitoes more frequently in the high temperature experiment to improve our statistical power of identifying differences among treatments.

For all surviving mosquitoes in each carton, we measured two components of vector competence, midgut infection and virus dissemination from the midgut in infected females, using a qualitative indirect fluorescence assay (Q-IFA). Virus EIP measurements were based on detection of a disseminated DENV infection in the mosquito.

We separated and tested bodies (comprising of the thorax and abdomen) for midgut infection and heads for disseminated infection, independently. Samples were placed into 1 mL viral transport medium (VTM; 77.2% low glucose DMEM, 18.5% heat-inactivated fetal bovine serum, 3.8% penicillin/streptomycin, and 0.15% gentamycin and nystatin) with approximately ten 2 mm glass beads (Fisher Scientific, Pittsburg, PA) in a screw-top plastic vial. Following collection, all samples were frozen at −80°C for later analysis by Q-IFA. We also collected the whole bodies (without separation of heads) of dead females daily and tested them for infection status. Results from analysis of dead mosquitoes were included in our survival analyses.

### Data analysis

All data was analyzed using JMP software, version 10 (SAS Institute Inc., NC). Vector competence was analyzed by nominal logistic regression of the infection or dissemination status as a full-factorial function of temperature and DPE, and carton nested within temperature and DPE. Records of survival for individual females exposed to the infectious blood meals were kept throughout the duration of the both experiments. Female survival was analyzed using a Kaplan-Meier (log-rank) analysis, with females that were sacrificed on scoring days right-censored. We tested for differences in survival curves between different temperature regimes and infection status of recently dead mosquitoes. We corrected for multiple comparisons between treatment groups for our logistic regression and Kaplan-Meier analyses using a Bonferroni correction.

### Fluorescent Focus Assay (FFA)

We used an infectious fluorescent focus assay [18] to titrate virus in blood meals offered to the mosquitoes. One-day old confluent monolayers of Vero (green monkey kidney) cells in 8-well chamber slides (Nunc, Rochester, NY) were inoculated with serial 10-fold dilutions of virus and blood meal samples. Dilutions were prepared in 2% FBS media in duplicate and inoculum was allowed to infect the cells for 1 hr at 37°C. A negative control (the media used for the dilutions) was included in all titrations. The overlay applied to the cells after the incubation was made of a 1∶1 mix of 2% FBS media∶carboxymethyl cellulose (CMC; 2% in PBS). We allowed 2 days for virus to replicate in the monolayer, then the media was removed and the cells washed carefully. In each washing step, PBS was added to cells three times, allowed to rest for 3–5 min, before PBS was again removed. The cells were fixed with 3.7% formaldehyde for 30 min, then washed and stained with 75 µL 1∶250 dilution of primary mouse anti-DENV monoclonal antibody (MAB8705; Millipore, MA) at 37°C for 1 hr. The cells were again washed to minimize background fluorescence, and then stained with 75 µL 1∶85 dilution FITC-conjugated secondary goat anti-mouse antibody (AP124F; Millipore, MA) for 30 min, which was used to detect and count the number of fluorescent foci under an FITC-fitted fluorescent microscope at 20× magnification.

### Qualitative Indirect Fluorescence Assay (Q-IFA)

To test mosquito samples for the presence of infectious DENV, we used a qualitative fluorescence assay. We homogenized the tissue samples for 4 min in a Retsch Mixer Mill 400, at 30 Hz. We filtered 300 µL of the sample through 0.22 µm cellulose acetate centrifuge filters (Costar Spin-X, Corning, Japan) and 50 µL of the filtered supernatant was inoculated in duplicate onto a 1-day old confluent monolayer of Vero cells, seeded at a density of 2.5×10^5^ cells/well in a 96-well culture plate. The inoculum was allowed to infect the cells for 1 hr at 37°C, before a standard maintenance media containing 2% FBS overlay was applied to the cells in each well, and the plate was incubated 37°C for 4 days. Positive and negative controls were used in each plate. We then removed the overlay, washed and fixed the cells in 3.7% formaldehyde for 20 min. The washing and staining steps that followed were exactly the same as for the FFA, except that the volumes used for antibody staining were 50 µL for each of the primary and secondary antibodies. We viewed cells under FITC-fitted fluorescence microscope at 10× to screen for the presence or absence of green fluorescence, which was indicative of a sample being either infected or uninfected by DENV, respectively.

## Results

### Vector competence

#### Low temperatures

**Figure 1:**
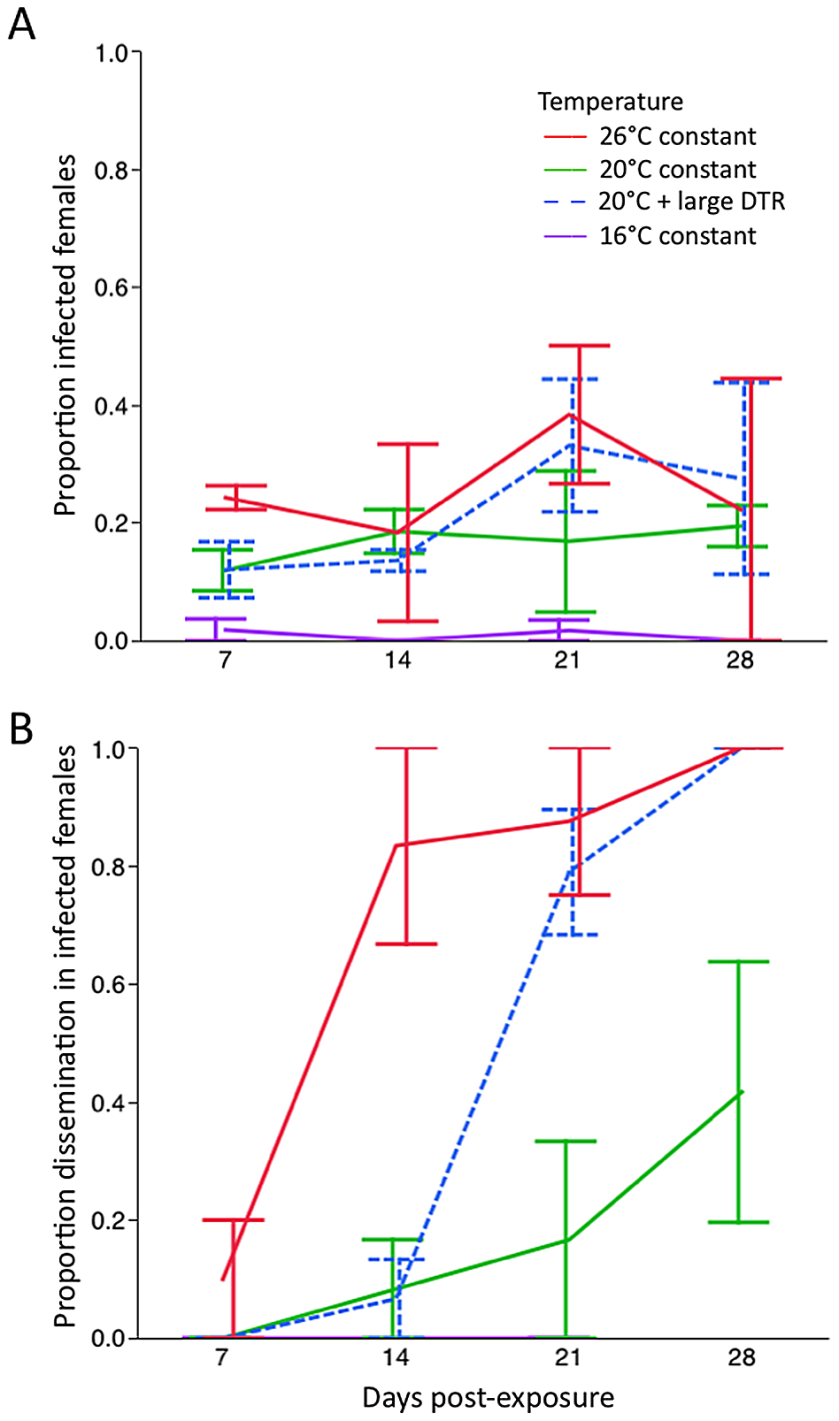
Proportion of *Ae. aegypti* with a detectable infection after being held at low temperatures. Females were held at 16°C, 20°C and 26°C constant, and 20°C with a large DTR and sampled at days 7, 14, 21 and 28 post-exposure to an infectious DENV-1 blood meal. A) Body infection, representing a detectable infection of the midgut tissue. B) Levels of infection in head tissue, representing a detectable disseminated infection.

A total of 769 female *Ae. aegypti* were sacrificed for testing vector competence at 7, 14, 21 and 28 DPE across four temperature regimes. The remaining 111 females died prior to sampling. There was a significant effect of temperature on body infection (χ^2^ = 15.068, df = 3, p = 0.0018; Figure 1A), however, pair-wise analyses show that the 16°C constant treatment had lower infection than all other treatments (p<0.01 in all cases). No other pairs were statistically different from each other (p>0.18 in all cases). The highest levels of infection were observed in mosquitoes exposed to 26°C constant (mean 25.8%), followed by 20°C + large DTR (25.6%) and 20°C constant (18.4%). There were only three individuals out of 240 from the 16°C treatment that were infected. EIP had no effect on infection (χ^2^<0.001, df = 3, p = 0.9999) in any low temperature treatment, and there was no interaction between temperature and EIP (χ^2^ = 6.008, df = 9, p = 0.739). There was, however, a significant effect of replicate carton nested within temperature and EIP (χ^2^ = 66.34, df = 28, p<0.0001).

Dissemination of DENV-1 to head tissue was tested on a total of 145 mosquitoes, 129 of which were sacrificed at the days indicated above. At 16°C, there were no individuals with an infected body that had a detectable disseminated infection and this temperature treatment was, therefore, excluded from subsequent analysis. For the remaining three temperatures, we found a significant effect of temperature at 14, 21 and 28 DPE, but not at 7 DPE (Table 1). There was no effect of carton nested within temperature at any incubation period tested.

**Table 1:** Temperature effects on dissemination probability of *Ae. aegypti* infected with DENV-1 exposed to low temperatures.

Time point	Factor	?^2^	df	*p*
Day 7	Temperature	<0.0001	3	1.0000
	Carton[Temperature]	1.2749	5	0.9375
Day 14	Temperature	18.4072	2	0.0001
	Carton[Temperature]	5.9605	5	0.3101
Day 21	Temperature	16.8531	3	0.0008
	Carton[Temperature]	5.2339	4	0.2641
Day 28	Temperature	14.2789	3	0.0025
	Carton[Temperature]	6.1889	3	0.1028

At each time point, the effect of temperature, and carton nested within temperature was analyzed for the 26°C and 20°C constant temperatures, and the 20°C cyclic treatment. 16°C constant was not included in tests because only single individuals with a detectable infection at 7 and 21 days post-exposure were identified, neither of which had a disseminated infection.

We tested for pair-wise differences between temperatures at each incubation period; differences are shown in Figure 1B. The first females with a disseminated infection (head tissue) were observed at 7 DPE, from the 26°C treatment. For 20°C + large DTR and 20°C constant, dissemination was first observed seven days later. Differences were detected between treatments at 14 DPE; mean dissemination at 26°C rose to over 80%, while dissemination levels were 7% and 8% for the fluctuating and constant 20°C temperature treatments respectively (p = 0.0002 for both comparisons). At 21 DPE, however, dissemination at 20°C + large DTR increased sharply to almost 80%, and was not different to the 26°C treatment (χ^2^<0.001, df = 1, p = 0.9982). The percent dissemination of the 20°C constant treatment increased only slightly (up to ∼17%) and was significantly lower than both 26°C constant and 20°C with large DTR (p = 0.0005 and p = 0.0017 respectively). At 28 DPE, 100% of the mosquitoes from the 26°C and 20°C + large DTR treatments tested positive for dissemination. This was significantly higher than for the 20°C constant treatment, in which ∼42% tested positive (pair-wise comparison with 26°C constant, p = 0.012, and for 20°C with fluctuations, p = 0.0004).

The time taken for 50% of infected individuals to complete the EIP (EIP_50_) was estimated using logistic regression of dissemination rates. All three estimates of EIP_50_ were significantly different from each other (χ^2^<40.04, df = 2, p<0.0001). The 20°C constant treatment had an estimated EIP_50_ of 29.6 days (95% confidence interval: 23.9–141.9), for 20°C with fluctuations it was 18.9 days (95% CI: 15.7–20.9), and for 26°C constant it was 11.1 days (95% CI: 5.9–15.1).

#### High temperatures

**Figure 2:**
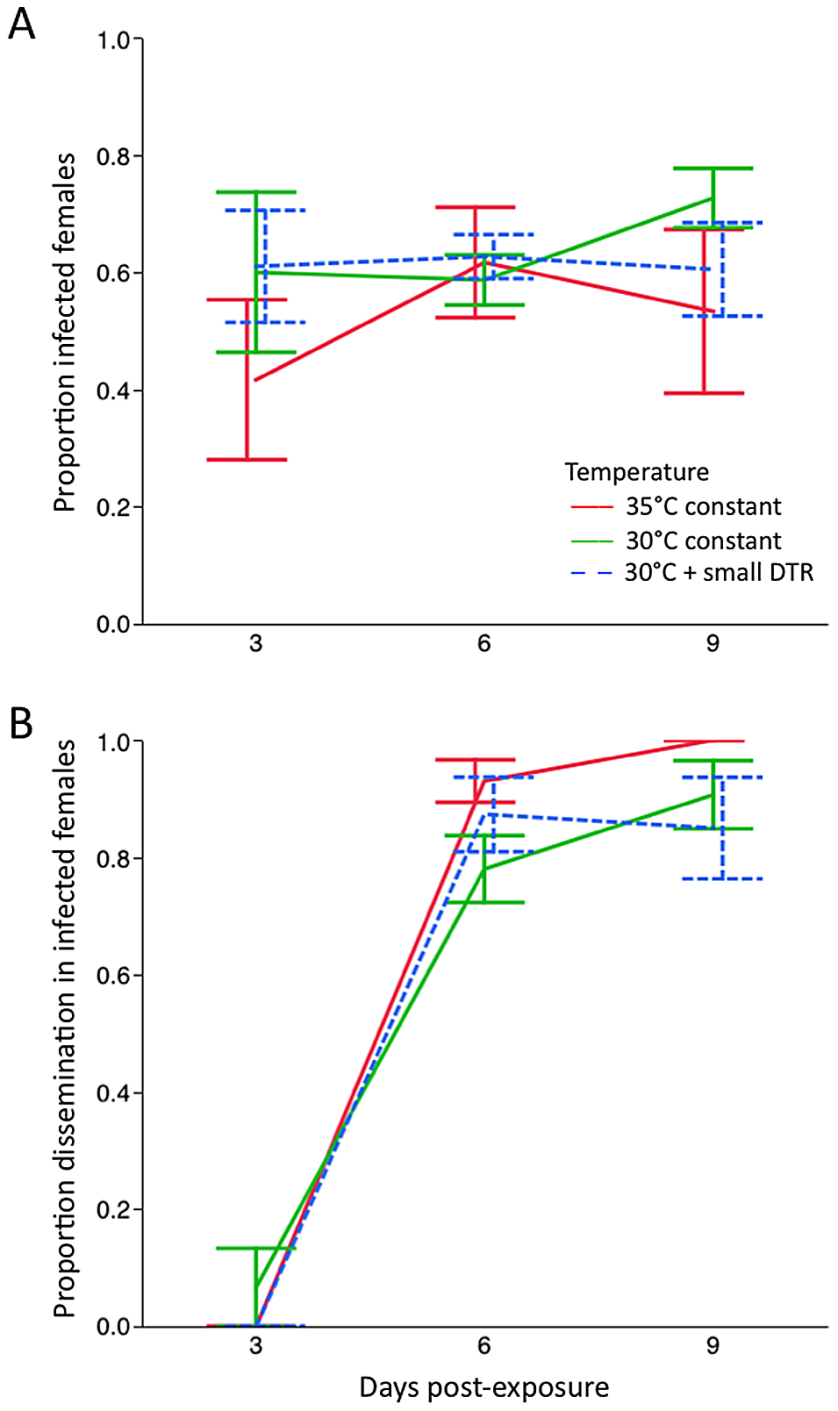
Proportion of *Ae. aegypti* with a detectable infection after being held at high temperatures. Females were held at 30°C and 35°C constant, and 30°C with a small DTR and sampled at days 3, 6 and 9 post-exposure to an infectious DENV-1 blood meal. A) Body infection, representing a detectable infection of the midgut tissue. B) Levels of infection in head tissue, representing a detectable disseminated infection.

We assessed the infection status of a total of 458 female *Ae. aegypti* bodies at 3, 6 and 9 days post exposure to an infectious DENV-1 blood meal. Average percent infection in the female bodies for each temperature treatment were 61% for 30°C + fluctuations, 64% for 30°C constant and 52% for 35°C constant. The percentage of infected females rose from 54% at day 3, to 61% for day 6 and 62% for day 9 (Figure 2A). We found no effect of temperature (χ^2^ = 0.496, df = 2, p = 0.084), EIP (χ^2^ = 1.72, df = 2, p = 0.423), or an interaction between these two factors (χ^2^ = 4.36, df = 4, p = 0.358). A significant effect of carton nested within temperature and EIP was observed (χ^2^ = 35.29 df = 19, p = 0.013).

A total of 251 head tissue samples were tested for dissemination. Three infected mosquitoes from the 30°C treatment that were found dead at 1 DPE tested negative for dissemination. The remaining 248 head tissue samples were tested after their collection at 3, 6 or 9 DPE. Temperature did not influence the proportion of infected females with a disseminated infection (χ^2^<0.0001, df = 2, p = 0.999). As expected dissemination increased sharply from 3 to 6 DPE (χ^2^ = 140.16 df = 2, p<0.0001). The earliest dissemination was observed in a single female collected 3 DPE from the 30°C constant treatment. By 6 and 9 DPE, all mosquitoes had 75–100% dissemination to their head tissue (Figure 2B). There was no statistical interaction between temperature and EIP (χ^2^<0.0001, df = 4, p = 0.999), and no effect of carton nested within temperature and EIP (χ^2^ = 0.212, df = 19, p = 0.325). There were no differences in EIP_50_ among any treatments (χ^2^ = 3.79, df = 2, p = 0.1498). We estimated EIP_50_ to be 5.16 days (95% CI: 4.7–5.6).

### Survival

#### Low temperatures

**Figure 3:**
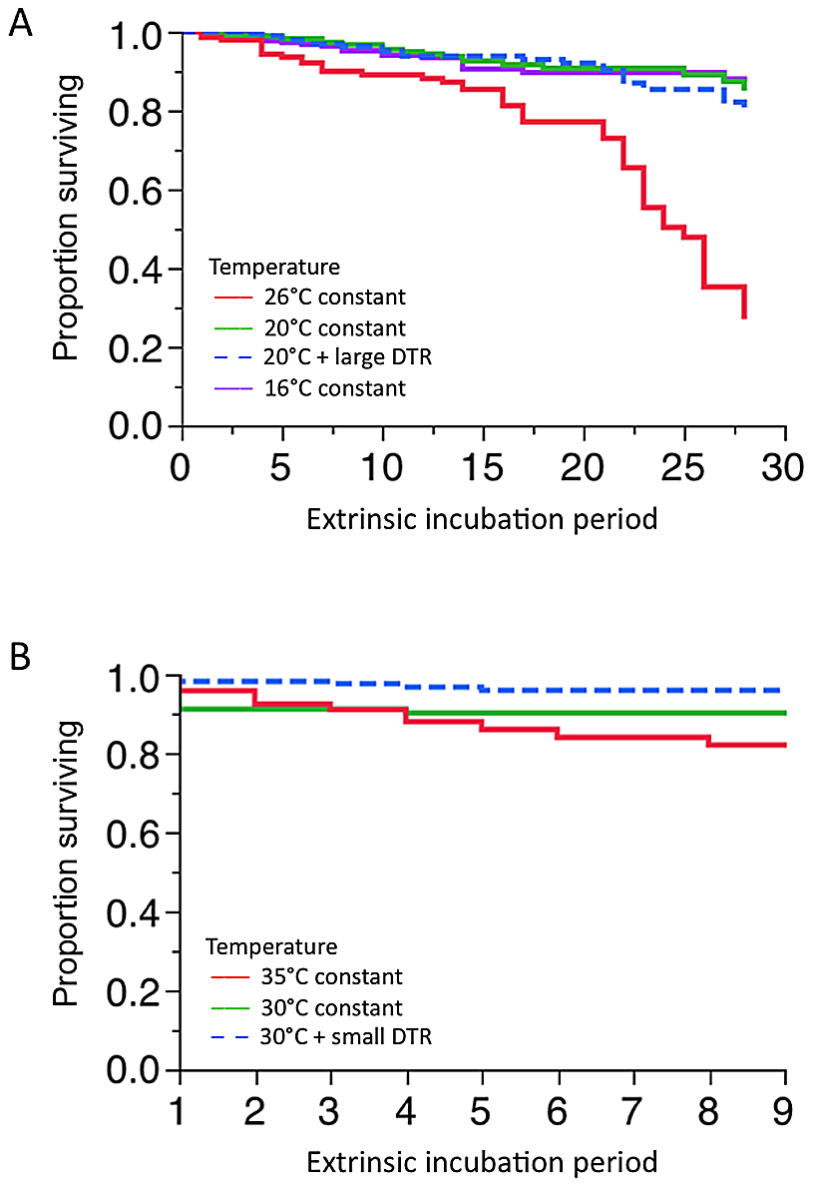
Survival of females exposed to DENV-1 from various constant and cyclic temperature regimes. A) Females held at low temperatures and a 26°C control. Despite the overall effect of temperature (p<0.0001), there were no differences in the survival curves after 28 days, between any of the three low temperature treatments (p>0.7). At 26°C constant, mortality was greater than in each of the low temperature treatments (p<0.001). B) Females held at high temperatures. Temperature influenced overall survival curves (p = 0.006), but only the curves of 30°C plus small DTR and 35°C constant were statistically different from each other (p = 0.001).

There were very low mortality rates for female *Ae. aegypti* exposed to DENV-1 at low temperatures during the 28 days of our experiment. Mean lifespan for each of the three low temperature treatments was 25 DPE at 16°C, and 26 DPE for both of the 20°C constant and fluctuating temperature treatments. At 26°C, mean lifespan was 22 DPE. Temperature influenced the survival curves of mosquitoes (χ^2^ = 74.87, df = 3, p<0.0001; Figure 3A), although pair-wise comparisons determined that this difference was due only to changes between the 26°C treatment with the other three treatments (p<0.0001 in all cases, still significant after a Bonferroni correction, where α = 0.0083). There were no differences between any of the remaining three treatments (p>0.7 for all comparisons). Infection status did not influence survival at 26°C constant, or either of the 20°C treatments (p>0.126 for all). At 16°C however, a total of three infected females were identified; one infected female was collected after it died at 4 DPE, the other two were censored (i.e., included in the analysis as being alive when sampled for vector competence). Due to the overall small number of infected females, the single death lead to a sharp, significant reduction in the survival rate in infected females to 66% by 4 DPE (χ^2^ = 4.195, df = 1, p = 0.0405).

#### High temperatures

Survival of females was followed for nine days under three high temperature treatments (30°C, 35°C and 30°C with small DTR). Females from the 35°C treatment had the highest mortality rates. There was a significant overall temperature effect (χ^2^ = 10.24, df = 2, p = 0.006, Figure 3B). Pair-wise comparisons show that only the 30°C plus small DTR and 35°C constant temperature treatments were statistically different from each other, there were no differences between other pairs. Only three infected females died throughout the entire experiment; all of these were from the 30°C constant treatment. Because of the low mortality rate of infected females, the relative mortality of uninfected females was greater (χ^2^ = 43.431, df = 1, p<0.001). In each treatment group, females had significantly higher survival when they had a detectable infection (p<0.0018 for all three temperatures).

## Discussion

Compared to a constant temperature, large diurnal temperature fluctuations at a mean of 20°C reduced the EIP_50_ for *Ae. aegypti* with a disseminated DENV-1 infection by approximately 36%, from 29.6 to 18.9 days. These results indicate a greater potential for DENV transmission at cool temperatures with natural fluctuations, and at an accelerated rate compared to what would be predicted by analysis of a 20°C constant temperature regime. Nevertheless, low intrinsic mortality under each of the low temperatures (those below 26°C) supports the potential for a mosquito to complete virus EIP at low temperatures, allowing for subsequent transmission following a protracted incubation period.

Females exposed to a large DTR around a 20°C mean were more likely to have detectable disseminated DENV-1 after 28 days compared to those reared under a constant, control temperature (100% *vs.* 41.7% dissemination). Whether fluctuations at 20°C also increased the maximum proportion of infected females with a disseminated infection compared to 20°C constant cannot be ascertained from our data. We did not see dissemination at the constant 20°C temperature plateau or reach maximal levels in our 28 day experiment. It is possible that dissemination levels could have reached 100% if we had held mosquitoes for a longer time. Regardless, the accelerated EIP under the cyclic temperature compared to the equivalent constant temperature indicates the potential for laboratory experiments using constant temperatures to significantly underestimate the duration of EIP in nature. Relatively low mortality rates under the three cooler temperatures (<20% after 28 days) compared to 26°C constant (∼30%) suggest that lifespan will not be a limiting factor in transmission potential during cooler times of the year or in more temperate environments. Epidemiologically, this substantial reduction in the EIP of DENV at low temperatures with fluctuations, in combination with low mortality rates, would be expected to increase vectorial capacity, and thus virus transmission potential, compared to constant temperatures. It would be useful in future experiments to improve temporal resolution by increasing sampling between the intervals we used, and allow mosquitoes at cooler constant temperatures longer to complete the EIP to identify maximum dissemination levels.

We observed a very low proportion of DENV-1 infected females held at 16°C constant. The youngest of the three infected females identified was found dead at 4 DPE, while the remaining two females were collected at 7 and 21 DPE during our weekly sampling. Due to slow digestion at such a low temperature, it is possible that the 4 and 7 DPE mosquitoes retained some infectious blood from the blood meal several days earlier. Although it is possible for a mosquito to become infected with DENV at 16°C, as shown by a single individual with a body infection at 21 DPE, this low temperature sharply reduced vector competence for DENV in *Ae. aegypti*. While we did not observe any mosquito with dissemination at 16°C, *Ae. aegypti* exposed to DENV and held at temperatures as low as 13°C for 32 days have previously been demonstrated to be capable of transmission [4]. We did not examine mosquitoes after 28 DPE and thus it is possible we did not allow enough time to observe transmission (as estimated by dissemination) under the 16°C treatment, and/or the mosquitoes used differed in their susceptibility to DENV infection [19], [20].

There was no detectable effect of the small fluctuations around a high mean of 30°C in the proportion of females with a midgut infection or disseminated virus, or in the duration of the EIP compared to the constant temperature control. The entire temperature profile (∼27°C to 35°C) falls within limits known to be highly conducive to DENV transmission, therefore, the lack of observable change is possibly due to the magnitude of the DTR not being large enough to produce a detectable response given our sample size. We did not test the large DTR around a mean of 30°C because there are few locations that we are aware of that have such large amplitude fluctuations at high temperatures. We therefore restricted our use of the large DTR to lower temperatures. Our cyclic low temperature treatment was derived from ambient conditions in dengue-endemic northern Thailand between December and January [21].

Results from previous studies indicate that midgut infection levels were lower under fluctuating temperature regimes with a mean of 26°C compared to constant temperatures, leading to reduced transmission potential [9]. Conversely, in the present study we observe that fluctuating temperatures at a lower mean lead to positive changes in the probability of virus dissemination from the midgut, consequently increasing transmission potential. Lambrechts et al. [9] predicted infection and dissemination probabilities of females infected with DENV and the duration of the EIP under various magnitudes of DTR. Their theoretical model predicted ∼50% of *Ae. aegypti* would become infected at both a constant 20°C and 20°C with large fluctuations. Although observed infection levels in our experiments under both temperature profiles were lower than that predicted we did not detect a statistical difference between these two temperature regimes, in agreement with the model. A mean of 18°C was predicted to be a pivotal mean temperature, above which fluctuations would decrease dissemination probability and below which they would enhance dissemination. Our results on dissemination rates imply that this predicted pivotal temperature rather lies between 20°C and 26°C. We hypothesize that the opposite effects of these two temperatures is due to differences in rates of viral growth/replication at different temperatures experienced by the mosquitoes. At a mean of 26°C, viral replication rates at the lower extreme of the temperature profile (∼18°C) might slow the virus more than it accelerates it at the upper end of the scale (∼36°C), resulting in a net deceleration compared to the rate at a constant 26°C. Conversely at a mean of 20°C, where replication is already slow, the low temperatures experienced by mosquitoes at the bottom of the fluctuating temperature profile lower the rate of replication to zero, but the relative increase in replication as the temperature rises to ∼30°C at the peak of the profile during the day will increase replication far more than it is decreased overnight, leading to a net acceleration.

Lambrechts et al. [9] did not model the effect of DTR above a mean of 28°C, although according to their predictions, small fluctuations are expected to result in close to a 100% midgut infection, and 80% dissemination, with a virus EIP shorter than 10 days. Observed dissemination results and estimates of EIP in our study are not in disagreement with this prediction, although again infection levels were lower. Midgut infection, dissemination and EIP estimates to produce the model were obtained from multiple experimental mosquito-flavivirus infections (not including DENV), and as a result, this discrepancy between the predictions and observed results may be a result of differences between vector-virus systems. The low infectious titers used in these two experiments are likely responsible for the low proportion of infected individuals obtained. Despite this, such titers fall within the reported range of viremia observed in humans [22], [23].

Although we used only a single serotype (DENV-1) to test the hypothesis that fluctuations at high and low mean temperatures would alter mosquito vector competence, the EIP of the virus, and adult survival, cumulative results from our group [9], [13] demonstrate consistency between results from similar experimental temperature regimes, despite using two mosquito populations, two serotypes (DENV-1 and DENV-2), two virus strains within one of these serotypes, and different infectious titers of the blood meals. We are therefore confident that the present study reveals another level of complexity in the interaction between the vector, viral pathogen and temperature.

Our results indicate that the effect of fluctuations around a low mean temperature markedly reduce EIP, which has important implications for determining DENV transmission risk at the northern and southern edges of DENV's geographic range, areas with a mean temperature that would normally be considered too low for DENV transmission to occur. Additionally, seasonal variation in DENV transmission, which is a common feature of DENV transmission dynamics [6], [24], can be associated with changes in mean temperature and DTR [9], [25], [26]. Conditions similar to the low temperature fluctuating profile used in this study (e.g., a mean below 22°C and DTR greater than 15°C), are observed in the low DENV transmission season throughout many parts of South East Asia, including areas in northern Thailand, Myanmar and central/northern India [21]. Each of these countries lie within the top 20 countries reporting the largest number of dengue cases annually [27], and despite low mean temperatures due to northern latitudes and often altitude, according to the World Health Organization, seasonal DENV transmission still occurs annually in such areas. Studies in *Anopheles stephensi* indicate a similar response to cyclic temperatures. A DTR at low temperatures enhances malaria transmission, while at higher temperatures equivalent DTRs reduced transmission potential [16]. Another recent study on arboviruses examined the interplay between temperature and EIP in *Culex pipiens* infected with West Nile virus [28], demonstrating that environmental conditions could enhance transmission of one variant over another. In this study however, realistic temperature fluctuations were not considered. An improved understanding of pathogen transmission across more realistic environmental conditions will allow for greater accuracy in modeling efforts to aid vector control and disease prevention in the future. It is, therefore, important that in future studies when researchers test mosquitoes at lower temperatures, realistic conditions are considered.

Similar responses to temperature changes have been reported for life-history trait estimates of *Ae. albopictus* and *Ae. aegypti* [10], [29]–[32]. It is likely that their responses to fluctuations in temperature would be comparable. *Ae. albopictus* often display a generalist blood feeding behavior [33], and is a competent vector of DENV [34]–[36]. Importantly, the species inhabits both tropical and temperate climates [37]. It is significantly more tolerant to cooler conditions than *Ae. aegypti* [38] and, therefore, poses a risk for arbovirus transmission in more temperate regions (e.g., Europe) [39]. As such, similar experiments on *Ae. albopictus* are warranted to better understand virus transmission potential in more temperate environments.

We observed limited mortality throughout the duration of both experiments, and identified females with a disseminated infection in six of the seven temperature treatments tested (all but 16°C). Mosquitoes were raised under conditions with optimal nutrition and were maintained in an environment with limited risk of death other than intrinsic factors and temperature. We do not know the maximum potential lifespan of mosquitoes exposed to each of these temperature regimes. We planned the experimental duration to be long enough for mosquitoes of each temperature to discern the duration of the EIP under each treatment, but did not attempt to estimate longevity. Epidemiologically, although these estimates represent a conservative estimate of the number of mosquitoes that might survive to such a time in order to transmit DENV, the high survival estimates compared to the duration of the EIP indicate that a relatively large proportion of infected mosquitoes in both experiments were capable under laboratory conditions of surviving to an age where they could transmit DENV to a susceptible host.

Similar to previous studies [40], [41], we observed reduced mortality in virus-infected females as opposed to those that were exposed, but uninfected. We observed this result, however, only at mean temperatures of 30°C or above. One hypothesis for this result is that mounting an immune response against the virus is more energetically costly than allowing the virus to establish infection [41]. Contrary to the results previously reported, we did not observe a significant difference between the survival of uninfected and infected females at low temperatures [40], [41]. This apparent interaction between temperature and infection could be due to the rapid proliferation of the virus at higher temperatures inducing a stronger immune response, where as at low temperatures, virus replication is slower and the immune response is, therefore, milder. Had we assessed survival for longer than 28 days, we may have detected a response when survival rates started to decline.

Our results indicate that the use of constant temperature experiments to assess *Ae. aegypti* vector competence for DENV at low temperatures underestimate the potential rate at which transmission may occur under more natural, fluctuating temperature profiles. Low intrinsic mortality at low temperatures with fluctuations similarly favors increased potential for virus transmission. Our results, therefore, provide a mechanism for sustained DENV transmission in endemic areas during cooler times of the year and indicate that transmission could be more efficient in temperate regions than previously anticipated.
